# Dorsal and Ventral Attention Systems: Distinct Neural Circuits but Collaborative Roles

**DOI:** 10.1177/1073858413494269

**Published:** 2014-04

**Authors:** Simone Vossel, Joy J. Geng, Gereon R. Fink

**Affiliations:** 1 Cognitive Neuroscience, Institute of Neuroscience & Medicine (INM-3), Research Centre Juelich, Germany; 2 Wellcome Trust Centre for Neuroimaging, University College London, UK; 3 Center for Mind and Brain and Department of Psychology, University of California Davis, USA; 4 Department of Neurology, University Hospital Cologne, Germany

**Keywords:** spatial attention, intraparietal sulcus, temporoparietal junction, spatial neglect, attentional networks

## Abstract

The idea of two separate attention networks in the human brain for the voluntary deployment of attention and the reorientation to unexpected events, respectively, has inspired an enormous amount of research over the past years. In this review, we will reconcile these theoretical ideas on the dorsal and ventral attentional system with recent empirical findings from human neuroimaging experiments and studies in stroke patients. We will highlight how novel methods—such as the analysis of effective connectivity or the combination of neurostimulation with functional magnetic resonance imaging—have contributed to our understanding of the functionality and interaction of the two systems. We conclude that neither of the two networks controls attentional processes in isolation and that the flexible interaction between both systems enables the dynamic control of attention in relation to top-down goals and bottom-up sensory stimulation. We discuss which brain regions potentially govern this interaction according to current task demands.

## Introduction

More than a decade ago, Corbetta and Shulman published their influential review article in which they introduced the concept of two anatomically and functionally distinct attention systems in the human brain (Corbetta and Shulman 2002). Broadly speaking, a dorsal frontoparietal system was proposed to mediate the top-down guided voluntary allocation of attention to locations or features, whereas a ventral frontoparietal system was assumed to be involved in detecting unattended or unexpected stimuli and triggering shifts of attention. Although the major nodes of the dorsal and ventral network—and many of their functional roles—are no longer debated, many critical questions remain. These outstanding issues concern the functional organization and hemispheric lateralization within each network, their specificity for attentional processes, and the interaction of the two networks with each other. The present review shall particularly focus on this latter aspect, that is, the interplay between the two networks for flexible attentional control. However, both networks will first be described separately in terms of their anatomy and functional specialization. Most of the work described will focus on the visuospatial attention system. It has been shown, however, that studies in other sensory modalities (such as audition and touch) reveal similar effects. This has led to the proposal that the dorsal and ventral networks are potentially supramodal attention systems (Macaluso 2010; Macaluso and Driver 2005).

## Functional and Structural Anatomy of the Dorsal and Ventral Attention Systems

**Figure 1.**
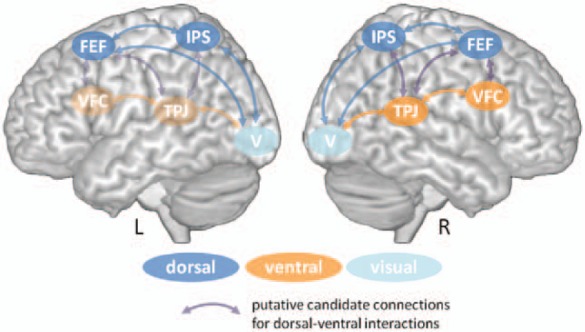
Schematic illustration of the components of the dorsal (blue) and ventral (orange) attention system in the human brain. Whereas there is evidence for a bilateral organization of the dorsal system, the ventral system might be more lateralized to the right hemisphere, although this assumption is challenged by recent neuroimaging data (see text for a further discussion of this issue). Putative intra- and internetwork connections are exemplarily depicted by bidirectional arrows. Interhemispheric connections between homologue areas are not shown. FEF = frontal eye fields; IPS = intraparietal sulcus; VFC = ventral frontal cortex; TPJ = temporoparietal junction; V = visual cortex.

The following paragraph shall outline the critical nodes of the dorsal and ventral attention network and describe their functional and structural anatomy. Figure 1 provides a schematic overview over the components of both systems as well as putative candidate connections for their interaction.

**Figure 2.**
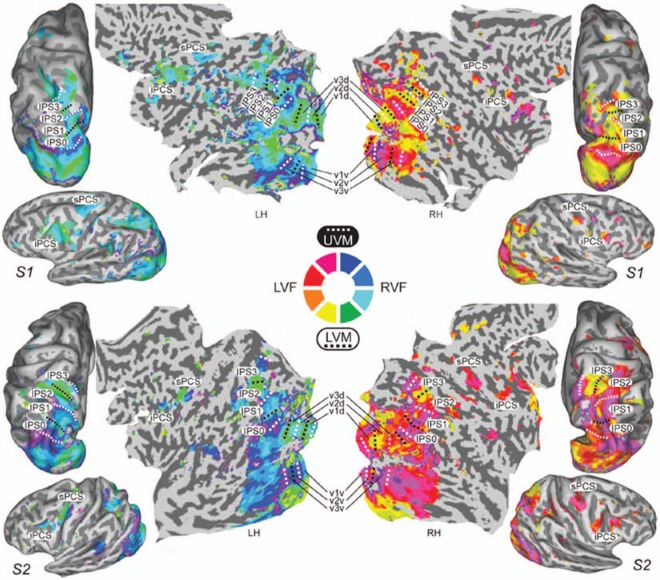
Topographic maps in visual, parietal, and frontal brain areas of two exemplary subjects from a study by Jerde and others (2012). UVM/LVM = upper/lower visual meridian; LVF/RVF = left/right visual field; LH/RH = left/right hemisphere; IPS = intraparietal sulcus; iPCS/sPCS = inferior/superior precentral sulcus. Reprinted with permission of the Society for Neuroscience, from Jerde and others (2012).

The dorsal network (Fig. 1, blue) is supposed to be organized bilaterally and comprises the intraparietal sulcus (IPS) and the frontal eye fields (FEF) of each hemisphere. These areas are active when attention is overtly or covertly oriented in space (e.g., after a predictive spatial cue [arrow] in Posner’s location-cueing paradigm; Posner 1980). Both IPS and FEF contain areas with retinotopically organized maps of contralateral space (Fig. 2; for a review, see Silver and Kastner 2009), which makes them candidate regions for the maintenance of spatial priority maps for covert spatial attention, saccade planning, and visual working memory (Jerde and others 2012). It has been proposed that the middle third of the IPS represents the human homologue of the monkey lateral intraparietal area LIP (Vandenberghe and Gillebert 2009). Interestingly, the dorsal frontoparietal network is also activated during feature-based attention (e.g., when the color of a target stimulus is precued) and provides a spatial coding in multiple reference frames (see Ptak 2012 for a comprehensive review).

The ventral network comprises the temporoparietal junction (TPJ) and the ventral frontal cortex (VFC) (Fig. 1, orange) and typically responds when behaviorally relevant stimuli occur unexpectedly (e.g., when they appear outside the cued focus of spatial attention). In contrast to the dorsal nodes (FEF and IPS) for which homologue areas are well described in nonhuman primates and which are hence well characterized with regard to their neuronal receptive field properties, the existence of homologue areas of the ventral regions is debated. So far, no standardized anatomical definitions exist for the localization of TPJ and VFC (see also Geng and Vossel unpublished data). Although the cytoarchitectonic parcellation of the posterior parietal cortex has recently been characterized (Caspers and others 2006) and can be used to specify the anatomical localization of fMRI activations, it has also been shown that functional activations do not clearly follow cytoarchitectonic boundaries (Gillebert and others 2013). Furthermore, TPJ might not be a single unitary structure but rather consist of multiple subregions with different connectivity patterns (Mars and others 2011; Mars and others 2012). To date no topographic maps in these ventral areas have been detected, although this might be because of methodological limitations of human neuroimaging experiments (Corbetta and Shulman 2011). However, spatial specificity for the contralateral hemifield has been observed for the right TPJ in a recent transcranial magnetic stimulation (TMS) study (Chang and others 2013).

It has been proposed that the ventral system is lateralized to the right hemisphere of the brain (Corbetta and Shulman 2002; Corbetta and others 2008). Whereas functional imaging studies indeed more consistently report right-hemispheric activation in temporoparietal areas, the left TPJ has also been shown to subserve attentional functions (DiQuattro and Geng 2011; Weidner and others 2009), and several studies have observed bilateral TPJ activation in tasks tapping attentional reorienting and the processing of rare deviant stimuli (Downar and others 2000; Geng and Mangun 2011; Serences and others 2005; Vossel and others 2009). A study by Doricchi and others (2010) found differences between left and right TPJ function, such that the left TPJ responded to invalidly as well as validly cued targets (as compared to trials with neutral cues) in a location-cueing paradigm, but the right TPJ showed higher activity for invalidly than validly cued targets.

**Figure 3.**
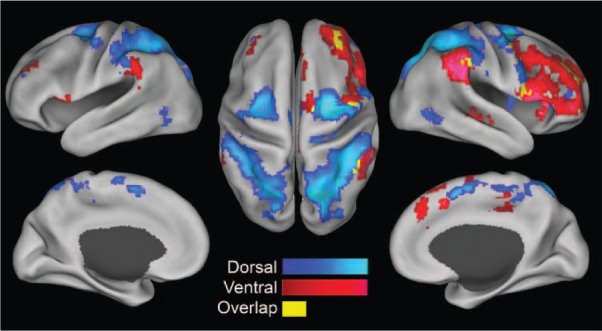
Functional connectivity maps for dorsal seed regions (IPS/FEF, blue) and ventral seed regions (TPJ/VFC, red) during fMRI resting state. Reprinted with permission of the National Academy of Sciences, USA, from Fox and others (2006).

Functional MRI studies looking at spontaneous (“resting-state”) functional connectivity between brain areas have shown that the dorsal and ventral networks are clearly distinguishable on the basis of their correlation patterns even under task-free conditions (see Fig. 3) (Fox and others 2006; He and others 2007). This inherent segregation of the two networks is also evident in their white matter structural connectivity. For example, Umarova and others (2009) used frontoparietal brain regions activated in a visuospatial attention task as seeds for probabilistic fiber tracking and found different fiber tracts with dorsal and ventral trajectories between them. Three major fiber tracts connect frontoparietal brain regions: the dorsal, middle, and ventral superior longitudinal fasciculi (SLF I, SLF II, and SLF III) (Thiebaut de Schotten and others 2011). Interestingly, there is evidence for a dorsal to ventral gradient of lateralization of the three SLF, and the degree of hemispheric lateralization is related to visuospatial behavioral performance (Thiebaut de Schotten and others 2011). Moreover, the connectivity patterns of left and right TPJ seem to be qualitatively different, with higher connectivity between TPJ and insula in the right hemisphere and higher connectivity between TPJ and the inferior frontal gyrus (IFG) in the left hemisphere (Kucyi and others 2012).

Taken together, the dorsal and ventral networks are two anatomically segregated cortical systems with functionally specialized nodes promoting specific processes for attentional control. It is so far unclear whether—and if so to what extent—functional asymmetries exist between the dorsal and ventral areas of each hemisphere, although there is evidence for such asymmetries in the ventral system. We will return to this issue below when we reconsider each system in more detail and discuss how interactions between the dorsal and the ventral network might be implemented in the human brain to enable a flexible deployment of attention.

## Top-Down Biases Emerging from the Dorsal System

It is now well recognized that the biasing of sensory areas (e.g., visual areas during the cue-induced expectation of a behaviorally relevant stimulus) emerges from higher-level areas in the frontoparietal cortex. Evidence for a crucial role of both IPS and FEF comes from functional imaging studies looking at the effective (i.e., causal or directed) connectivity between frontoparietal and sensory regions, as well as from studies combining fMRI with TMS.

Effective connectivity can be studied with analysis approaches such as dynamic causal modeling (DCM) (Friston and others 2003) or Granger causality (Roebroeck and others 2005). Studies investigating effective connectivity within the dorsal network have shown that IPS and FEF exert top-down influences on visual areas during the spatial orienting of attention. Using Granger causality analyses, Bressler and others (2008) demonstrated that both IPS and FEF influence the activity in visual areas in a top-down manner and that these influences are greater than the reverse bottom-up effects from visual cortex. A second study employing DCM has shown that directed influences from left and right IPS to left and right visual cortex are modulated by the direction of spatial attention in a “push-pull” fashion and cause a biasing of neural activity in visual areas (Vossel and others 2012). This finding is in accordance with the observation that the current locus of attention can best be decoded by interhemispheric differences of neural activity (Sylvester and others 2007).

Besides investigating connectivity patterns between brain areas, the combination of TMS and fMRI provides a valuable technique to study the causal impact of TMS applied over a target region exerted on other remote brain areas (for a review, see Driver and others 2010). In a series of studies, concurrent TMS of the FEF or IPS has been employed to investigate the neurostimulation effects on BOLD responses in visual areas (Ruff and others 2006; Ruff and others 2008; Ruff and others 2009). Paralleling the findings from effective connectivity fMRI studies, this work has demonstrated a significant modulation of visual cortex activity after both FEF and IPS TMS. However, in contrast to right IPS TMS, the effects of right FEF TMS differ for central and peripheral retinotopic visual areas (Ruff and others 2006; Ruff and others 2008). Moreover, the effects of right-hemispheric stimulation are more substantial then for left-hemispheric stimulation and are mostly observed in bilateral visual areas (Ruff and others 2009). Interestingly, the effects of parietal TMS are further modulated by the current attentional state (Blankenburg and others 2010). Although these findings do not allow for conclusions about the dorsal network architecture per se (i.e., the directness or indirectness of the stimulation effects), they for the first time provided causal evidence for the emergence of bias signals of visual cortex in FEF and IPS in humans (see Moore and Armstrong [2003] for original work on FEF microstimulation in monkeys). These results are complemented by an fMRI study in patients with selective lesions in the intraparietal area (Vuilleumier and others 2008). Here, it was shown that right IPS lesions lead to an asymmetric activation of retinotopic visual areas by task-irrelevant checkerboards. Interestingly, this effect was only present under high attentional load at fixation, thus highlighting the dynamic and state-dependent organization of the (visuo-)spatial network.

**Figure 4.**
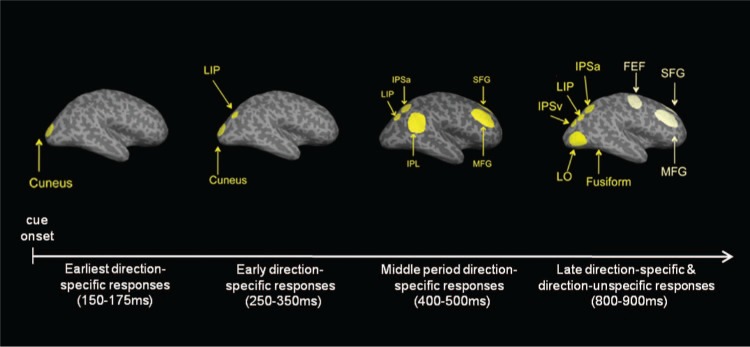
Direction-specific and -unspecific responses after the presentation of a spatial attention cue in different regions of interest and time intervals after cue onset. LIP = lateral intraparietal area; IPSa = anterior intraparietal sulcus; IPL = inferior parietal lobe; SFG = superior frontal gyrus; MFG = middle frontal gyrus; IPSv = ventral intraparietal sulcus; FEF = frontal eye fields. Reprinted with permission of the Society of Neuroscience, from Simpson and others (2011).

The investigation of the timing of responses in the different network nodes with methods offering a higher temporal resolution than fMRI (i.e., magnetoencephalography [MEG] or electroencephalography [EEG]) has provided further insights into the functionality of the dorsal system. A recent MEG study by Simpson and others (2011) examined the time course of direction-specific and direction-unspecific responses in several regions of interest after the onset of a centrally presented spatial cue that oriented attention to the left or right hemifield (see Fig. 4). The results showed early direction-specific responses in the cuneus and parietal areas, with direction-unspecific responses occurring later in time in frontal areas. Studies looking at oscillatory activity rather than event-related responses/fields moreover suggest that the involvement of the different regions at different time points is frequency-specific. In particular, visual and parietal areas show activity in the alpha and beta frequency bands in the cue-target period, whereas the appearance of the target stimulus is associated with a subsequent gamma band response (Siegel and others 2008). This finding is in line with the recent proposal that lower frequencies in the alpha and beta range mediate top-down (feedback) effects, whereas bottom-up (feedforward) effects involve gamma band activity (Bastos and others 2012).

In sum, recent research has clearly demonstrated that dorsal frontoparietal areas can causally modulate the activity of visual areas. However, the concurrent TMS fMRI studies challenge the view of strictly symmetrical functions of left and right IPS and FEF. Moreover, during spatial orienting of attention direction-specific responses can be found in the dorsal attention network, but these might critically depend on the time period after the onset of the spatial cue and hence may remain undetected by methods with low temporal resolution such as fMRI. It should further be noted that dorsal frontoparietal areas are also activated during feature-based attention (Liu and others 2011), where attention is not directed to a particular location in space, and more research is needed to reveal the differential neural bases of these two attentional mechanisms (see however Schenkluhn and others 2008; Serences and Boynton 2007).

## Reorienting Responses and Filtering in the Ventral System

The ventral attention system is typically recruited by infrequent or unexpected events that are behaviorally relevant (e.g., invalidly cued targets in the Posner task or oddballs). For this reason, this network has been implicated in stimulus-driven attentional control (Corbetta and Shulman 2002). During top-down guided attentional processing such as visual search, or under high visual short-term memory load, the activity in ventral areas such as TPJ is suppressed (Shulman and others 2003; Shulman and others 2007; Todd and others 2005). This has been interpreted as a filtering mechanism during a focused state of attention to protect goal-driven behavior and visual short-term memory content from irrelevant distractors. These top-down signals most likely originate in dorsal regions such as IPS and FEF, which are active during visual search (Shulman and others 2003). Interestingly, the deactivation of TPJ changes into an activation when salient nontargets in a visual search array carry information about the target stimulus (contextual cueing; Geng and Mangun 2011). Analysis of effective connectivity has moreover shown that this behavior crucially depends on dorsal–ventral interactions (DiQuattro and Geng 2011; see next section).

Human neuroimaging studies have shown that activation of TPJ can be observed across a variety of different cognitive domains such as attention, social cognition (“theory of mind”), and episodic memory. This has led to more generic interpretations of the role of this area in cognition. For instance, it has been proposed that TPJ might be generally involved in switching between different networks (Corbetta and others 2008). Another hypothesis is that the ventral parietal cortex relates to bottom-up attentional processing, which can be triggered not only by external sensory stimuli but also by internal memory-based information (Cabeza and others 2012). Moreover, the common involvement of TPJ in various domains might reflect a general role of this region for contextual updating (Geng and Vossel unpublished data). Hence, TPJ might not exclusively be involved in attentional reorienting and distractor filtering. Instead, the specific functions might rather depend on the connectivity of TPJ with other regions. Different subregions of TPJ are connected with different areas of the rest of the brain (Mars and others 2012), which might explain the involvement of TPJ complex in multiple domains. In what follows, we will specifically focus on the significance of these network interactions between ventral and dorsal frontoparietal areas in relation to flexible attentional control.

## Dorsal–Ventral Interactions

**Figure 5.**
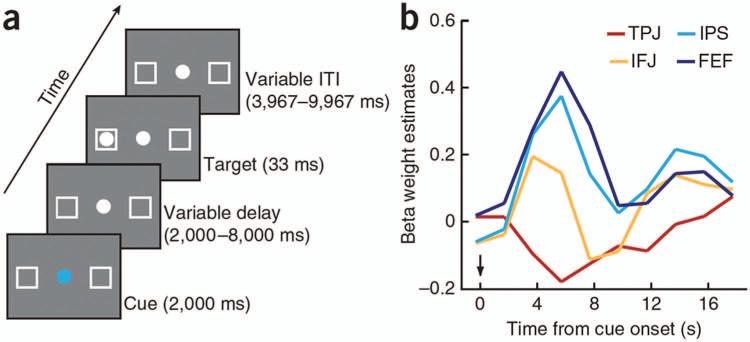
Illustration of the response in IPS, FEF, IFJ, and TPJ during the voluntary orienting of attention in the study by Asplund and others (2010). IPS = intraparietal sulcus; FEF = frontal eye fields; IFJ = inferior frontal junction; TPJ = temporoparietal junction. Reprinted with permission of Macmillan Publishers Ltd: Nature Neuroscience, from Asplund and others (2012).

This last section shall now focus on the interplay between the dorsal and ventral attention systems during attentional processing. To date, it remains to be established which areas or pathways provide the interface between both systems. The initial assumption of Corbetta and Shulman (2002) that TPJ acts as a circuit-breaker for the dorsal network is difficult to perpetuate (Corbetta and others 2008; Geng and Vossel unpublished data). One of the main reasons for this concerns the latencies of visual responses, which are shorter for IPS and FEF than for TPJ (for a discussion of this issue, see Corbetta and others 2008). In fact, a recent study using single cell recordings in monkeys has shown that early visual responses in the FEF are already correlated with perception and suggested that this fast activity might not be inherited from visual areas (Libedinsky and Livingstone 2011). These findings suggest that the TPJ plays a role in the later evaluation of sensory events with regard to top-down expectations rather than sending an early reorienting signal to dorsal regions. Besides the TPJ, the right posterior middle frontal gyrus (MFG) has been discussed as another candidate region for linking the dorsal with the ventral system (Corbetta and others 2008). This proposal was motivated by functional connectivity studies of spontaneous fMRI activity, which observed that activity in the right MFG correlated with the activity of both attention networks (Fox and others 2006; He and others 2007). Moreover, functional resting-state connectivity between right MFG and TPJ has been shown to be correlated with functional connectivity between left and right IPS in acute neglect (He and others 2007). Another study investigated the involvement of the dorsal and ventral attention network in surprise-induced blindness (Asplund and others 2010). Surprise-induced blindness describes a transient deficit in visual awareness after the foveal presentation of an unexpected task-irrelevant stimulus. The results from this study suggested that the inferior frontal junction (IFJ) (i.e., the cortex at the posterior end of the inferior frontal sulcus) might provide the interface between both systems. The activity pattern in the IFJ was more similar to dorsal frontoparietal regions than to the TPJ, which showed a distinct response profile during both surprise-induced blindness as well as orienting to an endogenous spatial cue (see Fig. 5). A study employing a combined location-cueing and oddball paradigm has moreover shown that the activity in this region at the posterior border of the inferior and middle frontal gyrus responds to the regularity of events: Activity in this area decreased when more validly cued standard targets were presented in succession. In contrast, the activity to an invalidly cued or deviant target was enhanced when more standard trials had been presented beforehand (Vossel and others 2011).

With regard to white matter fiber tracts, the SLF I connects dorsal frontoparietal areas and the SLF III connects ventral frontoparietal regions (Thiebaut de Schotten and others 2011). The SLF II, however, connects the parietal component of the ventral network with the prefrontal component of the dorsal network and might hence provide a crucial communication pathway for the two systems (Thiebaut de Schotten and others 2011). Damage to the SLF II has been shown to be the best predictor for spatial neglect (see below) (Thiebaut de Schotten and others 2012).

Having discussed potential hubs and pathways connecting both networks, we will proceed to review findings that highlight the collaborative roles of both circuits.

Chica and others (2011) used TMS over the right IPS and TPJ to interfere with the orienting of attention after a spatial cue. Importantly, this study used two different cueing conditions. In the exogenous cueing condition, attention was cued by nonpredictive peripheral cues, while these cues predicted the location of the target with 67% cue validity in the endogenous cueing condition. Applying TMS over the right IPS interfered with both types of attentional orienting. In contrast, TMS of the right TPJ interfered with orienting in the exogenous condition only. These findings already highlight that dorsal and ventral networks seem to work in concert to promote specific attentional processes and that top-down or bottom-up processing cannot uniquely be attributed to one system in isolation.

**Figure 6.**
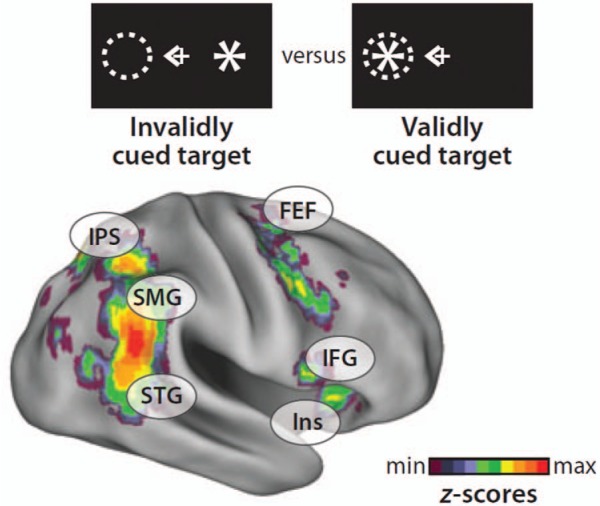
Coactivation of ventral and dorsal areas during reorienting of visuospatial attention. The depicted statistical map is based on a meta-analysis of four studies. IPS = intraparietal sulcus; FEF = frontal eye fields; SMG = supramarginal gyrus; STG = superior temporal gyrus; IFG = inferior fronal gyrus; Ins = insula. Reprinted with permission of Annual Reviews, Inc, from Corbetta and Shulman (2011).

Along the same lines, it is noteworthy that activation differences between invalid and valid trials (i.e., reorienting-related responses) in Posner’s location-cueing task are mostly observed in both dorsal and ventral frontoparietal areas (Corbetta and Shulman 2011; see Fig. 6).

This is not surprising because (as discussed above) there is only weak evidence that ventral areas contain spatial maps that are needed to reorient attention in space. Interestingly, whereas both networks are activated in conjunction during attentional reorienting, the opposite effect is observed during visual search where activation of the dorsal network is accompanied by a deactivation in the ventral network (Shulman and others 2003; Shulman and others 2007) (see Table 1). These results demonstrate the dynamic and flexible coupling of both systems in relation to current cognitive demands. This aspect is further highlighted by a study looking at Granger causality measures between dorsal and ventral networks and its impact on behavioral performance (Wen and others 2012). In this fMRI study, subjects were asked to respond to target stimuli in the cued hemifield and to ignore all stimuli in the uncued hemifield. Effective connectivity was analyzed for the time series of right TPJ and right IPS, as well as for the average of all regions of interest within the dorsal and ventral networks. Stronger Granger causal influences from right TPJ to right IPS were associated with higher response times and lower accuracy. In contrast, stronger influences from IPS to TPJ were accompanied with better behavioral performance. The same pattern was observed when the connectivity results were averaged across areas of the dorsal and ventral system.

**Table 1.** Overview of Findings from Different Experimental Paradigms Assessing Top-Down and Stimulus-Driven Attentional Processes in Relation to Activation of Dorsal and Ventral Frontoparietal Areas.

	IPS/FEF	TPJ	MFG/IFG
Top-down, goal-driven attention Orienting after symbolic predictive cues Visual search Visual short-term maintenance	↑	↓	↑/↓*
Stimulus-driven attention to salient behaviorally relevant events Orienting to exogenous cues Reorienting to unexpected events Response to contextual cues	↑	↑	↑

* Whereas one study reported activation of the MFG/IFG during orienting of voluntary attention (Asplund and others 2010), the MFG/IFG has been shown to be deactivated during top-down guided visual search (Shulman and others 2003). IPS = intraparietal sulcus; FEF = frontal eye fields; TPJ = temporoparietal junction; MFG = middle frontal gyrus; IFG = inferior fronal gyrus.

As noted above, the suppression of TPJ activity during top-down guided visual search is not observed in contextual cueing-paradigms, in which irrelevant stimuli carry predictive information about the target stimulus. DiQuattro and Geng (2011) observed that left TPJ and left IFG responded to the contextual relevance of nontarget stimuli and concluded that this signal is translated into an attentional control signal through interaction with dorsal regions. Using DCM, the authors showed that the FEF inhibited the TPJ when no informative stimuli were present. This finding demonstrates that dorsal regions filter information to the ventral network and that this mechanism indeed relies on interactive processes between dorsal and ventral regions such as FEF and TPJ. Moreover, this study shows that left-hemispheric areas and circuits also subserve attentional functions, hence further questioning the proposed strict right-lateralization of the ventral network.

Further valuable insights into dorsal–ventral interactions come from lesion or neuroimaging studies in brain-damaged patients. One of the most prominent impairments of attention after stroke is the spatial neglect syndrome, in which patients fail to orient and respond to events occurring in contralesional space (Halligan and others 2003). As this failure cannot be attributed to basic sensory-motor deficits alone, spatial neglect is often regarded as a disorder of spatial attention. Neglect symptoms can occur in all sensory modalities. The overall clinical manifestation of neglect resembles a disruption of the dorsal attention system, because the patients show a lateralized impairment in exploring and orienting to events in contralesional space. At the same time, neglect patients have difficulties in reorienting their attention to unexpected contralesional events (Friedrich and others 1998). Although neglect can be caused by lesions to many different cortical or subcortical brain areas, lesions to inferior parietal areas (Mort and others 2003) or the white matter lying underneath (Thiebaut de Schotten and others 2012) have most consistently been associated with neglect. In other words, neglect is more commonly observed after structural damage to parts of the ventral (and not the dorsal) system (Corbetta and Shulman 2011). However, a neuroimaging study in stroke patients has shown that the structural damage of ventral areas is accompanied by a functional impairment in the dorsal network. Corbetta and others (2005) investigated neglect patients with Posner’s location cueing task in the acute and recovered phases. They also employed fMRI in their patients to investigate activity changes in the two attentional systems. The presence of neglect in the acute stage was accompanied by an activity imbalance in dorsal parietal regions with a hyperactivation of left and a hypoactivation of right parietal areas. A similar pattern was observed for visual cortex. The activity imbalances were significantly related to neglect-related behavior in the Posner task and ameliorated with recovery from neglect. Moreover, a disruption of functional connectivity between parietal areas was characteristic for the presence of neglect (He and others 2007). Although the specificity of the hemispheric activity imbalance for spatial neglect has been questioned by another fMRI study in acute stroke patients (Umarova and others 2011), these data highlight how the damage in one system affects the functionality in structurally intact remote networks. Moreover, it has been shown that altering the activity in these structurally intact but functionally impaired regions with noninvasive brain stimulation techniques can ameliorate the spatial bias in patients with neglect (see, e.g., Sparing and others 2009).

Besides a failure to orient voluntarily to contralesional space, neglect patients show pronounced difficulties in reorienting attention to invalidly cued contralesional targets when attention needs to be disengaged from locations on the ipsilesional side and this reorienting deficit has originally been linked to lesions of the TPJ (Friedrich and others 1998). However, a recent neuroimaging study in patients with selective IPS lesions again challenges this simplified view and further emphasizes the notion that flexible attentional control relies on both dorsal and ventral mechanisms (Gillebert and others 2011). In this study, it was observed that attentional reorienting towards invalidly cued targets can be impaired after selective damage to the IPS. Similar to patients with inferior parietal brain damage, the two patients with IPS lesions showed a pronounced performance decrement when contralesional targets had been preceded by an invalid cue. Whether the deficit was strictly lateralized or not depended on the exact location of the lesion along the IPS. Most important, functional neuroimaging revealed that this impairment could not be attributed to functional impairments of the ventral attention system. One possible explanation for this finding is that attentional reorienting relies on both dorsal and ventral systems (see also Fig. 6). This notion is further supported by a DCM fMRI study in healthy subjects, which showed a modulation of connectivity from TPJ to IPS during reorienting of spatial attention (Vossel and others 2012).

## Conclusions

Although both dorsal and ventral attention systems are specialized for distinct attentional subprocesses such as top-down controlled attentional selection and the detection of unexpected but behaviorally relevant stimuli, respectively, it becomes obvious that flexible attentional control can only be implemented by dynamic interactions of both systems. Recent research has shown that this interaction pattern is flexible and crucially depends on the current task demands. Hence, activity in both systems can either be correlated or anticorrelated. There is evidence that this interplay is governed by frontal areas such as the inferior and middle frontal gyrus. The hemispheric functional specialization of each system and of their interaction needs to be addressed by future studies.

## References

[bibr1-1073858413494269] Asplund CL Todd JJ Snyder AP Marois R 2010 A central role for the lateral prefrontal cortex in goal-directed and stimulus-driven attention. Nat Neurosci 13(4):507–12 20208526 10.1038/nn.2509 PMC2847024

[bibr2-1073858413494269] Bastos AM Usrey WM Adams RA Mangun GR Fries P Friston KJ 2012 Canonical microcircuits for predictive coding. Neuron 76(4):695–711 23177956 10.1016/j.neuron.2012.10.038 PMC3777738

[bibr3-1073858413494269] Blankenburg F Ruff CC Bestmann S Bjoertomt O Josephs O Deichmann R, and others. 2010 Studying the role of human parietal cortex in visuospatial attention with concurrent TMS-fMRI. Cereb Cortex 20(11):2702–11 20176690 10.1093/cercor/bhq015 PMC2951847

[bibr4-1073858413494269] Bressler SL Tang W Sylvester CM Shulman GL Corbetta M 2008 Top-down control of human visual cortex by frontal and parietal cortex in anticipatory visual spatial attention. J Neurosci 28(40):10056–61 18829963 10.1523/JNEUROSCI.1776-08.2008 PMC2583122

[bibr5-1073858413494269] Cabeza R Ciaramelli E Moscovitch M 2012 Cognitive contributions of the ventral parietal cortex: an integrative theoretical account. Trends Cogn Sci 16(6):338–52 22609315 10.1016/j.tics.2012.04.008 PMC3367024

[bibr6-1073858413494269] Caspers S Geyer S Schleicher A Mohlberg H Amunts K Zilles K 2006 The human inferior parietal cortex: cytoarchitectonic parcellation and interindividual variability. NeuroImage 33(2):430–48 16949304 10.1016/j.neuroimage.2006.06.054

[bibr7-1073858413494269] Chang CF Hsu TY Tseng P Liang WK Tzeng OJ Hung DL, and others. 2013 Right temporoparietal junction and attentional reorienting. Hum Brain Mapp 34(4):869–77 22419442 10.1002/hbm.21476 PMC6870429

[bibr8-1073858413494269] Chica AB Bartolomeo P Valero-Cabré A 2011 Dorsal and ventral parietal contributions to spatial orienting in the human brain. J Neurosci 31(22):8143–9 21632936 10.1523/JNEUROSCI.5463-10.2010 PMC6622880

[bibr9-1073858413494269] Corbetta M Kincade MJ Lewis C Synder AZ Sapir A 2005 Neural basis and recovery of spatial attention deficits in spatial neglect. Nat Neurosci 8:1603–10 16234807 10.1038/nn1574

[bibr10-1073858413494269] Corbetta M Patel G Shulman GL 2008 The reorienting system of the human brain: from environment to theory of mind. Neuron 58(3):306–24 18466742 10.1016/j.neuron.2008.04.017 PMC2441869

[bibr11-1073858413494269] Corbetta M Shulman GL 2002 Control of goal-directed and stimulus-driven attention in the brain. Nat Rev Neurosci 3:201–15 11994752 10.1038/nrn755

[bibr12-1073858413494269] Corbetta M Shulman GL 2011 Spatial neglect and attention networks. Annu Rev Neurosci 34:569–99 21692662 10.1146/annurev-neuro-061010-113731 PMC3790661

[bibr13-1073858413494269] DiQuattro NE Geng JJ 2011 Contextual knowledge configures attentional control networks. J Neurosci 31(49):18026–35 22159116 10.1523/JNEUROSCI.4040-11.2011 PMC6634143

[bibr14-1073858413494269] Doricchi F Macci E Silvetti M Macaluso E 2010 Neural correlates of the spatial and expectancy components of endogenous and stimulus-driven orienting of attention in the Posner task. Cereb Cortex 20(7):1574–85 19846472 10.1093/cercor/bhp215

[bibr15-1073858413494269] Downar J Crawley AP Mikulis DJ Davis KD 2000 A multimodal cortical network for the detection of changes in the sensory environment. Nat Neurosci, 3:277–83 10700261 10.1038/72991

[bibr16-1073858413494269] Driver J Blankenburg F Bestmann S Ruff CC 2010 New approaches to the study of human brain networks underlying spatial attention and related processes. Exp Brain Res 206(2):153–62 20354681 10.1007/s00221-010-2205-7 PMC2940032

[bibr17-1073858413494269] Fox MD Corbetta M Snyder AZ Vincent JL Raichle ME 2006 Spontaneous neuronal activity distinguishes human dorsal and ventral attention systems. PNAS 103(26):10046–51 16788060 10.1073/pnas.0604187103 PMC1480402

[bibr18-1073858413494269] Friedrich FJ Egly R Rafal RD Beck D 1998 Spatial attention deficits in humans: a comparison of superior parietal and temporal-parietal junction lesions. Neuropsychology 12(2):193–207 9556766 10.1037//0894-4105.12.2.193

[bibr19-1073858413494269] Friston KJ Harrison L Penny W 2003 Dynamic causal modelling. NeuroImage 19(4):1273–302 12948688 10.1016/s1053-8119(03)00202-7

[bibr20-1073858413494269] Geng JJ Mangun GR 2011 Right temporoparietal junction activation by a salient contextual cue facilitates target discrimination. NeuroImage 54(1):594–601 20728548 10.1016/j.neuroimage.2010.08.025 PMC2993878

[bibr21-1073858413494269] Gillebert CR Mantini D Peeters R Dupont P Vandenberghe R 2013 Cytoarchitectonic mapping of attentional selection and reorienting in parietal cortex. NeuroImage 67:257–72 23201362 10.1016/j.neuroimage.2012.11.026

[bibr22-1073858413494269] Gillebert CR Mantini D Thijs V Sunaert S Dupont P Vandenberghe R 2011 Lesion evidence for the critical role of the intraparietal sulcus in spatial attention. Brain 134(6):1694–709 21576110 10.1093/brain/awr085

[bibr23-1073858413494269] Halligan PW Fink GR Marshall JC Vallar G 2003 Spatial cognition: evidence from visual neglect. Trends Cogn Sci 7(3):125–33 12639694 10.1016/s1364-6613(03)00032-9

[bibr24-1073858413494269] He BJ Snyder AZ Vincent JL Epstein A Shulman GL Corbetta M 2007 Breakdown of functional connectivity in frontoparietal networks underlies behavioral deficits in spatial neglect. Neuron 53:905–18 17359924 10.1016/j.neuron.2007.02.013

[bibr25-1073858413494269] Jerde TA Merriam EP Riggall AC Hedges JH Curtis CE 2012 Prioritized maps of space in human frontoparietal cortex. J Neurosci 32(48):17382–90 23197729 10.1523/JNEUROSCI.3810-12.2012 PMC3544526

[bibr26-1073858413494269] Kucyi A Moayedi M Weissman-Fogel I Hodaie M Davis KD 2012 Hemispheric asymmetry in white matter connectivity of the temporoparietal junction with the insula and prefrontal cortex. PLoS One 7(4):e35589 22536413 10.1371/journal.pone.0035589 PMC3334912

[bibr27-1073858413494269] Libedinsky C Livingstone M 2011 Role of prefrontal cortex in conscious visual perception. J Neuroscience 31(1):64–9 10.1523/JNEUROSCI.3620-10.2011 PMC3079255 21209190

[bibr28-1073858413494269] Liu T Hospadaruk L Zhu DC Gardner JL 2011 Feature-specific attentional priority signals in human cortex. J Neurosci 31(12):4484–95 21430149 10.1523/JNEUROSCI.5745-10.2011 PMC6622917

[bibr29-1073858413494269] Macaluso E 2010 Orienting of spatial attention and the interplay between the senses. Cortex 46(3):282–97 19540475 10.1016/j.cortex.2009.05.010

[bibr30-1073858413494269] Macaluso E Driver J 2005 Multisensory spatial interactions: a window onto functional integration in the human brain. Trends Neurosci 28(5):264–71 15866201 10.1016/j.tins.2005.03.008

[bibr31-1073858413494269] Mars RB Jbabdi S Sallet J O’Reilly JX Croxson PL Olivier E, and others. 2011 Diffusion-weighted imaging tractography-based parcellation of the human parietal cortex and comparison with human and macaque resting-state functional connectivity. J Neurosci 31(11):4087–100 21411650 10.1523/JNEUROSCI.5102-10.2011 PMC3091022

[bibr32-1073858413494269] Mars RB Sallet J Schüffelgen U Jbabdi S Toni I Rushworth MFS 2012 Connectivity-based subdivisions of the human right “temporoparietal junction area”: evidence for different areas participating in different cortical networks. Cereb Cortex 22(8):1894–903 21955921 10.1093/cercor/bhr268

[bibr33-1073858413494269] Moore T Armstrong KM 2003 Selective gating of visual signals by microstimulation of frontal cortex. Nature 421(6921):370–73 12540901 10.1038/nature01341

[bibr34-1073858413494269] Mort DJ Malhorta P Mannan SK Rorden C Pambakian A Kennard C, and others. 2003 The anatomy of visual neglect. Brain 126:1986–97 12821519 10.1093/brain/awg200

[bibr35-1073858413494269] Posner MI 1980 Orienting of attention. Q J Exp Psychol 32:3–25 7367577 10.1080/00335558008248231

[bibr36-1073858413494269] Ptak R 2012 The frontoparietal attention network of the human brain: action, saliency, and a priority map of the environment. Neuroscientist 18(5):502–15 21636849 10.1177/1073858411409051

[bibr37-1073858413494269] Roebroeck A Formisano E Goebel R 2005 Mapping directed influence over the brain using Granger causality and fMRI. NeuroImage 25(1):230–42 15734358 10.1016/j.neuroimage.2004.11.017

[bibr38-1073858413494269] Ruff CC Bestmann S Blankenburg F Bjoertomt O Josephs O Weiskopf N, and others. 2008 Distinct causal influences of parietal versus frontal areas on human visual cortex: evidence from concurrent TMS-fMRI. Cereb Cortex 18(4):817–27 17652468 10.1093/cercor/bhm128 PMC2601025

[bibr39-1073858413494269] Ruff CC Blankenburg F Bjoertomt O Bestmann S Freeman E Haynes JD, and others. 2006 Concurrent TMS-fMRI and psychophysics reveal frontal influences on human retinotopic visual cortex Curr Biol 16(15):1479–88 16890523 10.1016/j.cub.2006.06.057

[bibr40-1073858413494269] Ruff CC Blankenburg F Bjoertomt O Bestmann S Weiskopf N Driver J 2009 Hemispheric differences in frontal and parietal influences on human occipital cortex: direct confirmation with concurrent TMS-fMRI. J Cogn Neurosci 21(6):1146–61 18752395 10.1162/jocn.2009.21097 PMC2667814

[bibr41-1073858413494269] Schenkluhn B Ruff CC Heinen K Chambers CD 2008 Parietal stimulation decouples spatial and feature-based attention. J Neurosci 28(44):11106–10 18971453 10.1523/JNEUROSCI.3591-08.2008 PMC6671486

[bibr42-1073858413494269] Serences JT Boynton GM 2007 Feature-based attentional modulations in the absence of direct visual stimulation. Neuron 55(2):301–12 17640530 10.1016/j.neuron.2007.06.015

[bibr43-1073858413494269] Serences JT Shomstein S Leber AB Golay X Egeth HE Yantis S 2005 Coordination of voluntary and stimulus-driven attentional control in human cortex. Psychol Sci 16:114–22 15686577 10.1111/j.0956-7976.2005.00791.x

[bibr44-1073858413494269] Shulman GL Astafiev SV McAvoy MP D’Avossa G Corbetta M 2007 Right TPJ deactivation during visual search: functional significance and support for a filter hypothesis. Cereb Cortex 17(11):2625–33 17264254 10.1093/cercor/bhl170

[bibr45-1073858413494269] Shulman GL McAvoy MP Cowan MC Astafiev SV Tansy AP D’Avossa G, and others. 2003 Quantitative analysis of attention and detection signals during visual search. J Neurophysiol 90(5):3384–97 12917383 10.1152/jn.00343.2003

[bibr46-1073858413494269] Siegel M Donner TH Oostenveld R Fries P Engel AK 2008 Neuronal synchronization along the dorsal visual pathway reflects the focus of spatial attention. Neuron 60(4):709–19 19038226 10.1016/j.neuron.2008.09.010

[bibr47-1073858413494269] Silver MA Kastner S 2009 Topographic maps in human frontal and parietal cortex. Trends Cogn Sci 13(11):488–95 19758835 10.1016/j.tics.2009.08.005 PMC2767426

[bibr48-1073858413494269] Simpson GV Weber DL Dale CL Pantazis D Bressler SL Leahy RM, and others. 2011 Dynamic activation of frontal, parietal, and sensory regions underlying anticipatory visual spatial attention. J Neurosci 31(39):13880–9 21957250 10.1523/JNEUROSCI.1519-10.2011 PMC3672245

[bibr49-1073858413494269] Sparing R Thimm M Hesse MD Küst J Karbe H Fink GR 2009 Bidirectional alterations of interhemispheric parietal balance by non-invasive cortical stimulation. Brain 132(11):3011–20 19528092 10.1093/brain/awp154

[bibr50-1073858413494269] Sylvester CM Shulman GL Jack AI Corbetta M 2007 Asymmetry of anticipatory activity in visual cortex predicts the locus of attention and perception. J Neurosci 27(52):14424–33 18160650 10.1523/JNEUROSCI.3759-07.2007 PMC6673462

[bibr51-1073858413494269] Thiebaut de Schotten M Dell’Acqua F Forkel SJ Simmons A Vergani F Murphy DGM, and others. 2011 A lateralized brain network for visuospatial attention. Nat Neurosci 14(10):1245–6 21926985 10.1038/nn.2905

[bibr52-1073858413494269] Thiebaut de Schotten M Tomaiuolo F Aiello M Merola S Silvetti M Lecce F, and others. 2012 Damage to white matter pathways in subacute and chronic spatial neglect: a group study and 2 single-case studies with complete virtual “in vivo” tractography dissection. Cereb Cortex 10.1093/cercor/bhs351 23162045

[bibr53-1073858413494269] Todd JJ Fougnie D Marois R 2005 Visual short-term memory load suppresses temporo-parietal junction activity and induces inattentional blindness. Psychol Sci 16:965–72 16313661 10.1111/j.1467-9280.2005.01645.x

[bibr54-1073858413494269] Umarova RM Saur D Kaller CP Vry MS Glauche V Mader I, and others. 2011 Acute visual neglect and extinction: distinct functional state of the visuospatial attention system. Brain 134(11):3310–25 21948940 10.1093/brain/awr220

[bibr55-1073858413494269] Umarova RM Saur D Schnell S Kaller CP Vry MS Glauche V, and others. 2009 Structural connectivity for visuospatial attention: significance of ventral pathways. Cereb Cortex 20(1):121–9 19406904 10.1093/cercor/bhp086

[bibr56-1073858413494269] Vandenberghe R Gillebert CR 2009 Parcellation of parietal cortex: convergence between lesion-symptom mapping and mapping of the intact functioning brain. Behav Brain Res 199(2):171–82 19118580 10.1016/j.bbr.2008.12.005

[bibr57-1073858413494269] Vossel S Weidner R Driver J Friston KJ Fink GR 2012 Deconstructing the architecture of dorsal and ventral attention systems with dynamic causal modeling. J Neurosci 32(31):10637–48 22855813 10.1523/JNEUROSCI.0414-12.2012 PMC3432566

[bibr58-1073858413494269] Vossel S Weidner R Fink GR 2011 Dynamic coding of events within the inferior frontal gyrus in a probabilistic selective attention task. J Cogn Neurosci 23(2):414–24 20146598 10.1162/jocn.2010.21441

[bibr59-1073858413494269] Vossel S Weidner R Thiel CM Fink GR 2009 What is “odd” in Posner’s location-cueing paradigm? Neural responses to unexpected location and feature changes compared. J Cogn Neurosci 21(1):30–41 18476756 10.1162/jocn.2009.21003

[bibr60-1073858413494269] Vuilleumier P Schwartz S Verdon V Maravita A Hutton C Husain M, and others. 2008 Abnormal attentional modulation of retinotopic cortex in parietal patients with spatial neglect. Curr Biol 18(19):1525–9 18848444 10.1016/j.cub.2008.08.072 PMC2863074

[bibr61-1073858413494269] Weidner R Krummenacher J Reimann B Müller HJ Fink GR 2009 Sources of top-down control in visual search. J Cogn Neurosci 21(11):2100–13 19199412 10.1162/jocn.2008.21173

[bibr62-1073858413494269] Wen X Yao L Liu Y Ding M 2012 Causal interactions in attention networks predict behavioral performance. J Neurosci 32(4):1284–92 22279213 10.1523/JNEUROSCI.2817-11.2012 PMC6796284

